# Sustained Oscillations of NF-κB Produce Distinct Genome Scanning and Gene Expression Profiles

**DOI:** 10.1371/journal.pone.0007163

**Published:** 2009-09-29

**Authors:** Myong-Hee Sung, Luigi Salvatore, Rossana De Lorenzi, Anindya Indrawan, Manolis Pasparakis, Gordon L. Hager, Marco E. Bianchi, Alessandra Agresti

**Affiliations:** 1 Laboratory of Receptor Biology and Gene Expression, National Cancer Institute, National Institutes of Health, Bethesda, Maryland, United States of America; 2 Chromatin Dynamics Unit, DIBIT, San Raffaele Scientific Institute, Milan, Italy; 3 Institute for Genetics, University of Cologne, Cologne, Germany; IBM Thomas J. Watson Research Center, United States of America

## Abstract

NF-κB is a prototypic stress-responsive transcription factor that acts within a complex regulatory network. The signaling dynamics of endogenous NF-κB in single cells remain poorly understood. To examine real time dynamics in living cells, we monitored NF-κB activities at multiple timescales using GFP-p65 knock-in mouse embryonic fibroblasts. Oscillations in NF-κB were sustained in most cells, with several cycles of transient nuclear translocation after TNF-α stimulation. Mathematical modeling suggests that NF-κB oscillations are selected over other non-oscillatory dynamics by fine-tuning the relative strengths of feedback loops like IκBα. The ability of NF-κB to scan and interact with the genome in vivo remained remarkably constant from early to late cycles, as observed by fluorescence recovery after photobleaching (FRAP). Perturbation of long-term NF-κB oscillations interfered with its short-term interaction with chromatin and balanced transcriptional output, as predicted by the mathematical model. We propose that negative feedback loops do not simply terminate signaling, but rather promote oscillations of NF-κB in the nucleus, and these oscillations are functionally advantageous.

## Introduction

In mammalian cells, various signals such as hormones, cytokines, and cell-cell interfaces elicit changes in gene expression mediated by inducible transcription factors. Many latent transcription factors also induce expression of genes that provide feedback loops upon their signaling pathways. The negative feedback genes are generally thought to functionally terminate the signaling action of the transcription factor. These feedback loops also create the potential for the transcription factor activity to oscillate between active and inactive states over hours [1], [2], [3]. However, the significance of such oscillations is unclear. Are periodic cycles a mere side effect of the pathway settling back to the basal state, or do they have a functional role in signaling?

**Figure 1:**
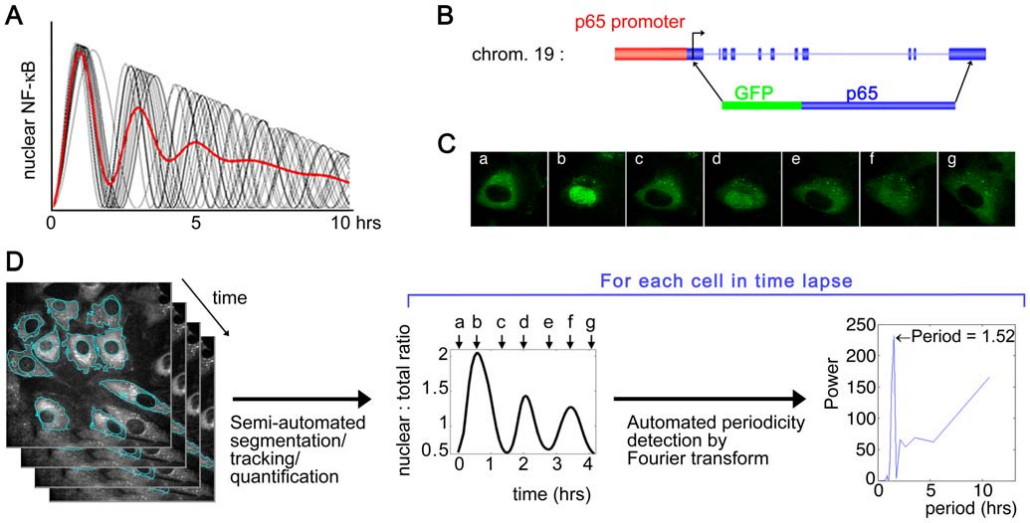
Real time monitoring of GFP-p65 using GFP knock-in MEF and live cell microscopy allows accurate quantification of single cell dynamics of endogenous p65. (A) The population average time course of nuclear NF-κB (red) can show strongly damped oscillation even when the individual cells (black, 20 out of 1000 shown) have sustained oscillatory dynamics. Thousand hypothetical sine waves were generated to have a period slightly varying from 2 hours (15% S.D. in cycle frequency) and linearly decreasing amplitude. The late peaks become unsynchronized, making the average profile appear constant. (B) The knock-in mice have the endogenous p65 locus replaced by GFP-p65 and have wild-type phenotype. (C) A typical time series of a single living cell treated with 10 ng/ml TNF-α and imaged overnight. The low GFP level required special image acquisition setup. The quantification of the GFP intensity data is shown in the time course plot in (D). (D) The time lapse image analysis procedure is shown schematically. The nuclear:total ratio of GFP-p65 plotted is for the cell in (C), where the labeled arrows correspond to the images. The ratio was obtained as the mean nuclear intensity divided by mean cellular intensity (see Methods). The periodogram on right has a single sharp peak and indicates that the estimated period is ∼1.5 hours for this cell.

NF-κB is a classical example of a transcription factor subject to negative feedback loops. NF-κB regulates numerous cell signaling processes and its activity is controlled in part by the level of nuclear translocation. In resting cells, the predominant dimer p65∶p50 exists mostly as a cytoplasmic complex bound to its inhibitor IκB proteins. Numerous upstream signals induce degradation of the IκB proteins following phosphorylation by the IκB kinase complex (IKK). This release from latency in the cytoplasm allows active NF-κB to translocate into the nucleus and activate expression of target genes, including several feedback genes [4]. One such target is the *Nfkbia* gene which codes for IκBα. The re-synthesis of IκBα acts as strong negative feedback, causing the inactivation of NF-κB and its re-localization to the cytoplasm. However, previous biochemical and genetic investigations have indicated that the propensity to oscillate, driven by induction of IκBα, is counteracted by mechanisms that modulate and dampen the oscillation [4], [5], [6], [7]. Observations at least from cell populations show rapid down-regulation of NF-κB after activation. This has led to the view that NF-κB signaling is biphasic, i.e. its activity rises rapidly and is strongly attenuated by feedback inactivation, and an oscillatory behavior after the first cycle of NF-κB activity might be insignificant or even deleterious for cellular function. However, the inherent limitation of kinetic studies using cell populations [1], [3], [8], [9] is that transient activities that are not synchronized from cell to cell cannot be detected even by the most quantitative assays (Fig. 1A) [10], [11].

Here, we report single-cell observations that show the prevalence of sustained oscillations in endogenous NF-κB localization. Using instant perturbations of the system and real-time monitoring, we show that the network dynamics can be altered as predicted by a mathematical model. We also observe a previously unsuspected nucleus-to-cytoplasm transport of active NF-κB. Through computational modeling and experimental evaluation, we explore the signaling roles of distinct NF-κB dynamics that occur in multiple timescales. Finally, we find that the fast interaction of NF-κB with chromatin remains the same from first to later cycles of nuclear localization, and the recurrent cycles contribute productively to the transcriptional output of the system.

## Results

### TNF-α induced oscillations of endogenous NF-κB are sustained in single cells expressing GFP-tagged p65 knocked into the native locus

To observe real time dynamics, we monitored the localization of NF-κB in single living cells, which are the basic unit for pathway operations and are the definitive context for probing pathway dynamics unambiguously. We used fibroblasts derived from knock-in mice where the endogenous p65 gene was replaced by GFP-p65 (Fig. 1B) [12]. This approach allows live cell imaging of the endogenous p65, without any of the potential problems associated with over-expressed proteins. The protein expression level of GFP-p65 in our knock-in fibroblasts was comparable to that in wild-type fibroblasts ([12] and data not shown). Activation profiles of p65 target genes in response to TNF-α were also very similar to wild-type cells ([12] and data not shown). Together with the normal phenotype of the knock-in mice [12], these data indicate that the GFP-p65 is functional and the NF-κB regulatory network is physiological. Since the expression of GFP-p65 was as low as the endogenous level, a microscope with a special optical scanner was necessary to visualize and quantify the GFP signal (see Materials and Methods, Fig. S1, Videos S1, S2).

**Figure 2:**
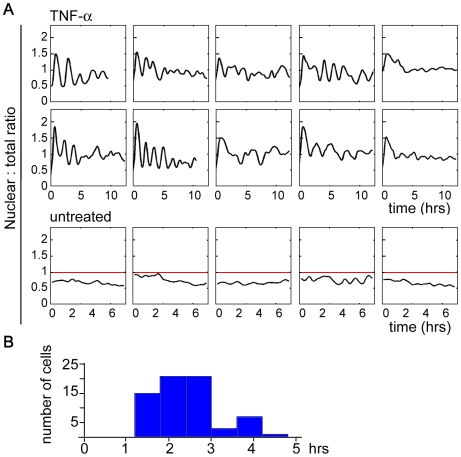
Periodic cycles in TNF-α induced NF-κB oscillations are sustained in most cells. (A) 79% of the cells (n = 95) had sustained oscillations in nuclear p65. Ten temporal profiles from TNF-α treated cells are shown, with eight oscillating (left 4 columns) and two non-oscillating (last column) cells. The lower panel is from control time lapse data where cells were followed by time lapse microscopy without TNF-α treatment. The red line at ratio 1 indicates the state where the nuclear and the total mean intensities are the same. (B) The distribution of the period identified by Fourier analysis (see Methods).

Figure 1 (C, center plot in D) shows an example of a time lapse experiment after TNF-α stimulation where we quantify the nuclear:total ratio of GFP-p65. The long-term dynamics of p65 in individual single cells ranged from 1∼2 cycles to dramatic oscillations with 6∼7 cycles. A minority of cells had a single cycle with subsequent minor and irregular fluctuations (8 oscillating cells and 2 non-oscillators are shown in Fig. 2A). Most cells showed multiple striking peaks; later peaks had gradually decreasing amplitudes but were clearly discernible. Often the p65 flux out of the nucleus between the periodic cycles was so efficient as to reduce the nuclear level to a basal state. We subjected the time course data from 95 cells to Fourier analysis (Fig. 1D) and found that the majority of the cells (79%) oscillated with a somewhat variable period with a median value of 2.2 hours (n = 75, Fig. 2B). Cells that were not treated with TNF-α had no nuclear translocation of p65 as expected (Fig. 2A bottom panel). Although we classified the time courses into two categories by the presence/absence of a strong periodic component, we cannot rule out the possibility that even the ‘non-oscillating’ cells may have weak oscillations which can nevertheless mediate some signaling effect.

### Computer simulations suggest that NF-κB oscillations are selected by fine-tuning network dynamics through the relative strengths of negative feedback loops

**Figure 3:**
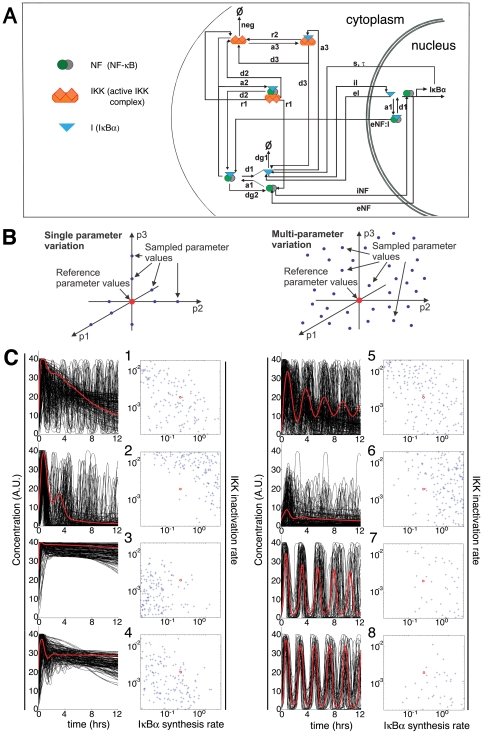
Computer simulations suggest that NF-κB dynamic profiles are mostly controlled by kinetic parameters for negative feedback. (A) A schematic representation of an NF-κB model. It includes essential regulatory events such as IKK activation, inducible/constitutive degradation of IκBα, nuclear import/export, inducible synthesis of IκBα, and post-stimulus attenuation of IKK activity. The quantitative model is described in full by the differential equations in Fig. S2A. (B) Two different approaches for parameter variation. Unlike single parameter variation, where one parameter is varied at a time from the reference value and the rest are fixed (top), we varied all parameter values simultaneously in a random fashion (bottom). (C) The dynamical model was numerically solved for a random sample of 1000 parameter combinations. Each kinetic parameter was varied by two orders of magnitude around the reference value (see Supplementary Information). From the simulated temporal profiles eight dominant patterns were extracted by K-means cluster analysis (K = 8). For each cluster, individual time courses of free nuclear p65 are shown in black, and a representative profile is in red. The underlying system parameter values (s, neg) that correspond to the simulations within the given cluster are presented to the right of each cluster plot. Only the two most influential parameters are plotted out of 18 co-varied parameters. The red dot indicates the reference parameter values.

To gain systems-level insight on the parameters that influence the observed NF-κB behavior in different cells after TNF-α stimulation, we explored variability and robustness properties of a minimal mathematical model that captures some essential pathway components (see Fig. 3A and Supplementary Information, Fig. S2) [13]. Parameter analysis is often done by varying one parameter at a time, fixing all the other parameter values. While computationally manageable, this results in an extremely limited investigation of the system properties [14] (Fig. 3B). Instead, we employed an extensive multi-parameter sampling approach, where a large set of random parameter combinations was chosen for model simulations. Exploration of a range of possible parameter values is necessary because of the uncertainty in the model parameter values that were inferred or compiled from reported experiments [15]. In fact, individual cells are likely to have slightly variable rate constants for each molecular process in the model.

Our computer simulations revealed several possible patterns of response for the NF-κB network after activation (Fig. 3C). Distinct patterns were readily identifiable when the K-means clustering algorithm was applied to group similar time courses. Numerous optimizations of K-means with different numbers of clusters indicated that these eight clusters were reproducible and representative. The patterns that closely represent the experimentally observed oscillatory profiles (clusters 5, 7, and 8, Fig. 3C; Fig. 2A, left 8 plots) were from parameter values that occupy a significantly broad region in the 18-dimensional parameter space. This implies that oscillation is a robust feature of this network.

Our multi-parameter variation analysis also shows a pattern with non-oscillating, one-peak responses (cluster 2), which is similar to the experimentally observed profile of non-oscillating cells (Fig. 2A, right 2 plots). This pattern resulted from parameter values for hyperactive IκBα induction and/or fast IKK inactivation. Thus, with an appropriate choice of feedback parameter values, the NF-κB system could operate in a non-oscillating mode, which may be embodied by the non-oscillating cells. The absence of some computationally predicted scenarios (clusters 1, 3, 4, and 6) from the experimentally observed time course profiles could be partly due to some unrealistic combinations of parameter values in our variation analysis. Alternatively, it might reflect a preference of the NF-κB regulatory network for oscillations, suggestive of functional advantage.

Next we extensively examined the correlation of these distinct responses with the underlying parameter values, and found that 16 out of 18 parameters had no clear influence on system dynamics (Fig. S2E), while inducible synthesis of IκBα and post-stimulus IKK inactivation are the major processes that determine the kinetic behavior (Fig. 3C). Notably, these are precisely the mechanisms that represent the two classes of negative feedback loops in the regulatory network. For example, low IκBα synthesis rate and/or persistent IKK activity explain the aberrant response pattern with no post-stimulus attenuation (cluster 3).

Taken together, observed dynamic patterns and modeling indicate that diverse behaviors, including non-oscillatory responses, are possible from the NF-κB network, and indeed a small fraction of cells do not oscillate. The above multi-parameter variations suggest that the relative strengths of the two classes of negative feedback is a strong determinant of NF-κB dynamics and that the oscillatory behavior represents a robust but selective pattern of the network dynamics. The prevalence of the oscillatory pattern, over other possible behaviors, suggests that it might be chosen for specific function and adaptive value, rather than imposed by the system design and thus inevitable.

### LMB or CHX co-treatment with TNF-α produces one cycle of nuclear NF-κB localization

Next we wished to investigate the functional role of NF-κB dynamics by altering the natural oscillations. Some commonly used perturbation methods, however, presented serious problems for our systems approach. For example, siRNA, widely used for specific down-regulation of genes, would be impossible to model at the single cell level because silencing action takes effect over a long time (∼hours) with its own kinetics, and cell-to-cell heterogeneity is invariably significant. Similarly, genetically disrupted cells settle into altered steady states long before the experiments by compensatory mechanisms, complicating model-based comparison of mutant behavior to that of wild-type. Thus, we chose to use nearly instantaneous inhibitors that interfere with key steps in the NF-κB network, using Leptomycin B (LMB), an inhibitor of nuclear protein export, and Cycloheximide (CHX), an inhibitor of protein synthesis.

**Figure 4:**
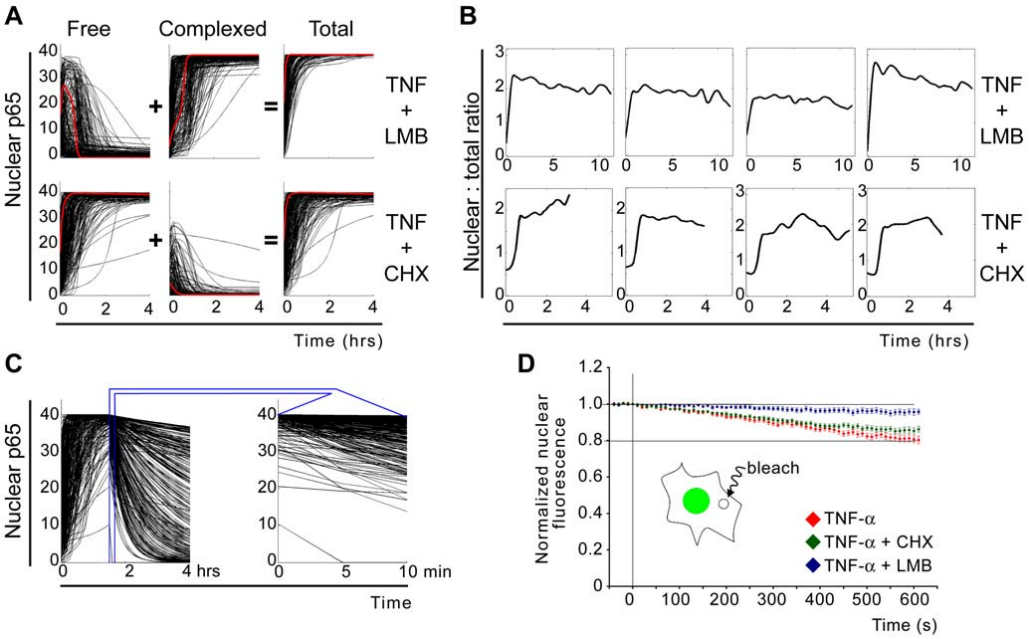
Two contrasting perturbations of NF-κB oscillations decouple nucleocytoplasmic shuttling from feedback-driven long-term dynamics. (A) Perturbed dynamics were simulated using the model in Fig. 3 and the parameter values from clusters 5, 7, and 8, with the additional modifications to nuclear export terms or the induced IκBα synthesis term for LMB or CHX effects, respectively (see text). The graphs show the time courses of free, IκBα-complexed, and total p65 in the nucleus, as predicted by the model. The red curves indicate the free and complexed components of a typical profile of nuclear p65. Inhibition of nuclear export allows one cycle of free p65 whereas inhibition of negative feedback induces constant activity. (B) Experimentally observed time lapse profiles of nuclear p65 level for single cells co-treated with LMB or CHX. Cells were treated with 10 ng/ml TNF-α and either 5 nM LMB or 2.5 µg/ml CHX, and followed by live cell imaging. (C) Nuclear export of free p65 was simulated using the model in Fig. 3 with the parameter values from clusters 5, 7, and 8, and s was reduced by 100-fold at t = 0 for the CHX effect. In addition, nuclear import parameters (iNF, iI) were reduced to 10^−5^ at t = 90 min after TNF-α and CHX co-treatment. The total nuclear p65 amount (which is close to the free p65 amount under this condition) is shown in the upper panel. The first 10 minute interval after inhibition of import is plotted on the expanded time axis on the right, to facilitate comparison to the experimentally measured time course in (D). (D) Fluorescence loss in photobleaching (FLIP) was performed to determine any ongoing exchange of p65 between the nucleus and the cytoplasm after its apparently complete nuclear translocation induced by TNF-α alone or in co-treatment with CHX or LMB. A circular spot in the cytoplasm was repeatedly bleached for 10 minutes while the nuclear mean intensity was monitored (inset). The relatively short timeframe for the FLIP protocol did not allow measurements up to complete loss of GFP signal. Error bars are S.E.

LMB blocks nuclear export of p65 into the cytoplasm, an enabling mechanism behind NF-κB oscillation. When cells are simultaneously treated with TNF-α and LMB, a single pulse of free nuclear NF-κB is expected to be followed by the formation of the NF-κB∶IκBα complex in the nucleus. This is also predicted by model simulations (Fig. 4A, upper panel; Parameter values were from cluster 5, 7, 8 in Fig. 3C, and all export rates were reduced by 10^5^-fold at t = 0; An example decomposition plot for TNF-α alone is shown in Fig. S2C for comparison). Time lapse imaging confirmed that total nuclear p65 accumulates in the nucleus (Fig. 4B, upper panel). The nuclear retention of IκBα-bound inactive NF-κB by LMB matches previous reports well [16].

On the other hand, co-treatment of CHX together with TNF-α inhibits IκBα re-synthesis, a dominant mechanism that drives the system dynamics toward oscillatory regimes. Live imaging showed that CHX constrains p65 in the nucleus for hours, as expected (Fig. 4B, lower panel). Nuclear p65 is predicted to be active (free of IκBα) under this condition (Fig. 4A, bottom panel; Parameter values were from cluster 5, 7, 8 in Fig. 3C, and s was reduced by 100-fold at t = 0).

### The two contrasting perturbations of NF-κB oscillations reveal reverse nucleus-to-cytoplasm flux

We noticed an unexpected difference between the time lapse imaging data from TNF-α only, TNF-α/LMB co-treated, and TNF-α/CHX co-treated cells: The temporal profiles of nuclear GFP-p65 in cells treated with TNF-α and LMB showed significantly higher peak translocation, in comparison to cells treated with TNF-α alone or with CHX (Fig. S3). This suggests the existence of the reverse-translocating (nucleus-to-cytoplasm) p65 molecules even during the early activation phase of NF-κB after TNF-α treatment, which had not been appreciated previously. It also implies that the nuclear p65 level at a given moment is simply the net result of the constituent fluxes.

We therefore asked whether p65 molecules may be exported from the nucleus independently of IκB. First, we ran model simulations with the IκBα synthesis rate set to zero (at t = 0), and also all nuclear import rates were reduced to zero when nuclear NF-κB level is maximal (at t = 90 min). If there was no export, blockade of nuclear import at this time would not have any effect on the nuclear p65 level. The simulation results showed gradual efflux of p65 into the cytoplasm that is appreciable within minutes for a wide range of parameter values (Fig. 4C). This was due to the slow export of free NF-κB that was allowed in the mathematical model, which was masked by the dominant IκBα-mediated export during the normal TNF-α induced dynamics (Fig. S2).

We then experimentally validated this somewhat neglected possibility by fluorescence loss in photobleaching (FLIP) (Fig. 4D). When cells are treated with TNF-α and CHX together, no IκB molecules are present because their re-synthesis is blocked after their TNF-α-dependent degradation. When p65 nuclear translocation was apparently complete (30 to 60 minutes after co-treatment), a cytoplasmic spot was bleached repeatedly. This caused a decrease in nuclear GFP-p65 signal, indicating the presence of p65 export into the cytoplasm. The observed time course is in agreement with the predicted loss of nuclear p65 (Fig. 4C), implying that NF-κB continuously exports out to the cytoplasm in an IκBα-independent manner. The retrograde flux of p65 was detectable only when the nuclear p65 level was maximal. When p65 was still accumulating in the nucleus 10 to 25 minutes after TNF-α and CHX, the same FLIP protocol did not produce a reduction in nuclear GFP-p65 level, most likely due to the import flux canceling out the loss by the export flux (Fig. S4). We note that a putative nuclear export signal within p65 has been reported [17].

The extent of FLIP in the presence of IκBα (TNF-α alone) was slightly faster than that observed in cells treated with TNF-α and CHX, which suggests that p65 export is indeed enhanced by IκBα (Fig. 4D). As a negative control, FLIP was applied to cells co-treated with TNF-α and LMB, which resulted in little or no reduction in nuclear GFP signal (Fig. 4D). These data indicate an inherent tendency of NF-κB to shuttle out to the cytoplasm independently of IκB. We also confirmed that IκBα increases p65 export efficiency upon TNF-α stimulation.

Taken together these results demonstrate that the nucleocytoplasmic shuttling of NF-κB is bi-directional and does not require oscillations or IκBα. This implies that NF-κB shuttles continuously, sampling the full intracellular environment, with or without concurrent oscillations in the level of nuclear p65.

### The characteristic genome-scanning activity of NF-κB is maintained through sustained oscillation

We predicted that the functional consequences of CHX and LMB perturbations would be extremely different on p65 activity, despite the similar effect on its nuclear localization. LMB would render NF-κB bound to IκBα, thus unable to bind chromatin, whereas CHX would maintain its ability to bind chromatin. To address this experimentally and compare perturbed conditions with the unperturbed behavior, we first evaluated features of the distinct p65 cycles in the nucleus using an assay with the temporal precision to resolve the asynchronous cycles in single cells. We note that a functional transcription factor scans the genome by binding and dissociating to chromatin repeatedly, and by diffusing within the nucleus [18]. This activity is indicated by a fast recovery of signal from the GFP fusion protein during fluorescence recovery after photobleaching (FRAP), a widely utilized assay. We recently discovered that functional NF-κB binds to target chromatin sites, with fast exchange kinetics on the timescale of seconds [19]. The chromatin residence time of p65, and its mobility measured by FRAP, are affected by its DNA binding affinity: a higher affinity mutant p65 has a slower mobility and lower affinity mutants have faster mobility [19], . Of note, the reported numbers of p65 molecules per cell and NF-κB binding sites in the genome are within an order of magnitude (60,000 [21] and 14,000 [19], [22], respectively). Such comparable numbers suggest that FRAP measurements from the nucleus of our GFP-p65 knock-in cells reflect some binding activity of endogenous p65 in living cells.

**Figure 5:**
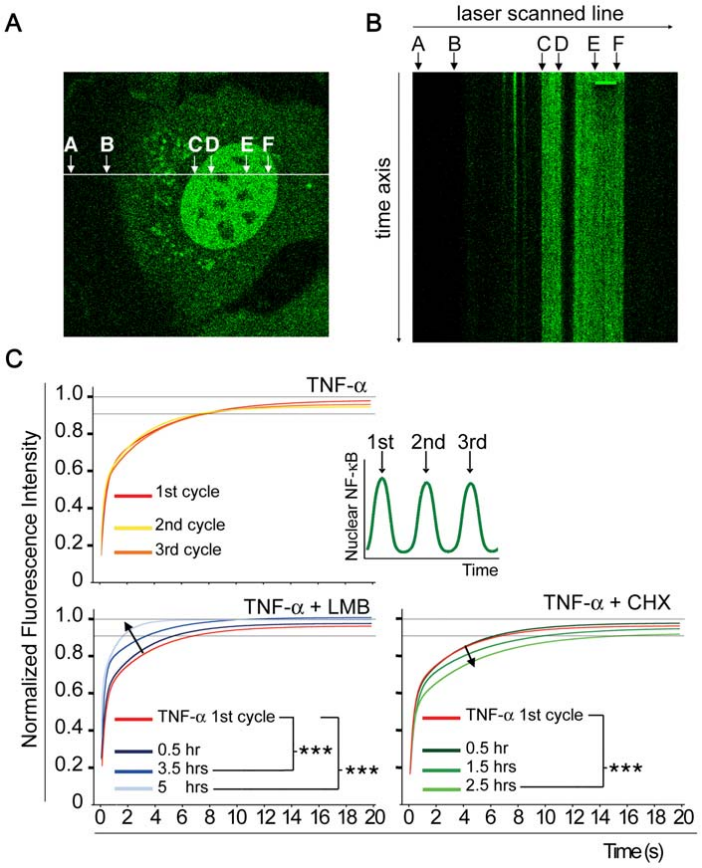
Perturbations of NF-κB oscillations by inhibiting either shuttling or IκBα re-synthesis cause opposite defects in characteristic genome-scanning activity of p65. (A) The line FRAP protocol. The line FRAP assay allowed photodamage-free in vivo analysis of the dim cells by limiting the laser scan area to a line. The white line is the scanning path of the confocal microscope. Segment EF represents the bleaching region (5 µm). Segment AB represents the background while CD is used to correct for the bleaching during imaging in the double normalization procedure (see Methods). This example shows a GFP-p65 knock-in cell co-treated with TNF-α and CHX for 2.5 hrs. In this condition, p65 mobility is the lowest (see panel B). (B) This image represents the line FRAP output from the cell in (A). The x axis is the laser scanning path. Each repetition of the line scanning appears as a line along the time axis. The bleached region is visible as a solid green rectangle. The dark area below the bleached region is indicative of the slow recovery. (C) Line FRAP experiments were performed at the specified times on cells treated with TNF-α (top panel) and on cells co-treated with TNF-α and LMB or CHX (bottom left and right panels). The recovery curves represent fitting of the raw data by bi-exponential curves and *** indicates a statistically significant difference (p<0.001) according to a permutation test (see Methods). The reference profile for all comparisons was that from the first p65 cycle after TNF-α (red curve, top panel). The arrows indicate the shift of the curves over time.

We used a non-invasive optimized line FRAP technique [23] (Fig. 5A, B) in conjunction with real time monitoring, and found that p65 mobility during peak of nuclear translocation did not change from early to late cycles of TNF-α induced oscillations (Fig. 5C, top panel). This result implies that p65 in the late translocation cycles is capable of diffusing on and interacting with the genome as effectively as the first p65 molecules activated after TNF-α. This is remarkable given the numerous feedback and post-translational mechanisms that act upon p65 to attenuate its activity following stimuli.

In contrast, when oscillations were blocked by either LMB or CHX, intranuclear p65 mobility measured by line FRAP was progressively altered with time. LMB induced faster mobility of GFP-p65, which is consistent with unproductive interaction of p65 with the chromatin (Fig. 5C, bottom left panel). CHX, however, effectively slowed down p65 mobility in the nucleus, indicative of prolonged binding of p65 with target chromatin and inefficient genome scanning (Fig. 5C, bottom right). These opposite outcomes are in line with the predicted differences in terms of free NF-κB. It is equally important to note that p65 mobility is still rather fast despite the absence of IκB in this condition, indicating that IκBα is not necessary for ‘displacing’ NF-κB from target promoters [24]. Our data suggest that p65 detaches from target chromatin without IκBα intervention, and IκBα might simply capture displaced p65, either in the nucleus or in the cytoplasm. Together with the FLIP data in Fig. 4D, the FRAP results support the idea that NF-κB has an inherent tendency to dissociate from the chromatin and shuttle out to the cytoplasm, as a feature of its dynamic behavior.

### NF-κB oscillations ensure a balanced gene expression program in response to TNF-α

**Figure 6:**
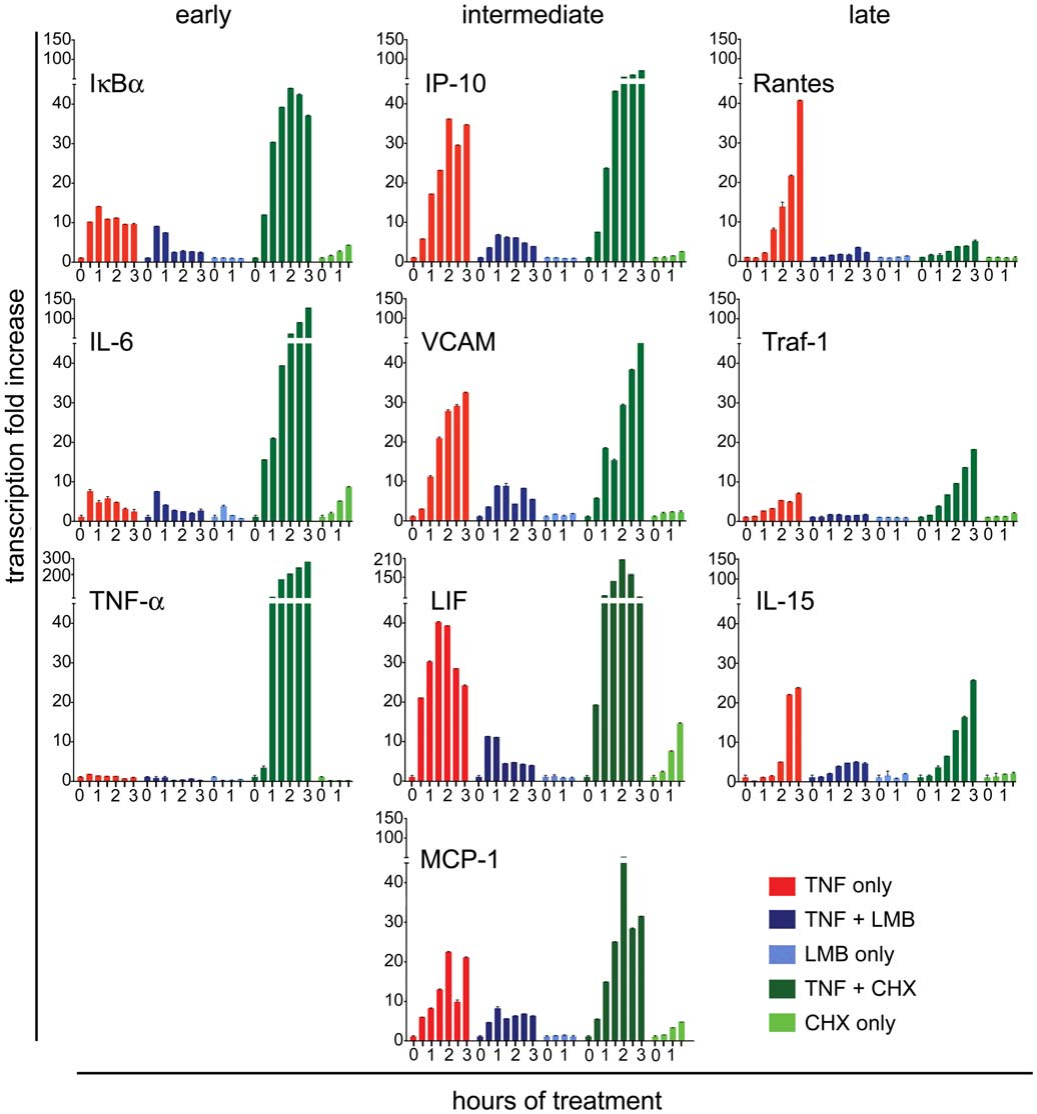
TNF-α induced gene expression programs are quantitatively modulated by perturbations of NF-κB dynamics that alter the number of signaling cycles or peak durations. Gene expression profiles were measured by q-PCR in cells stimulated as specified in the bottom right panel. RNA samples were prepared at half-hour intervals up to 3 hrs after stimulation as indicated on the time axis. Time-points for LMB- and CHX- only controls were up to 1.5 hrs.

The dynamical systems analysis and the mobility data from line FRAP suggest that even the late periodic cycles of NF-κB oscillations may contribute significantly to the functional response induced by TNF-α. To assess the role of oscillations on transcription, we measured by qRT-PCR the gene expression kinetics of representative NF-κB targets. ‘Early’ genes were maximally transcribed within half an hour after TNF-α stimulation, while ‘intermediate’ and ‘late’ primary target genes were activated later and highly expressed only after 1.5 and 3 hours, respectively (Fig. 6).

LMB co-treatment with TNF-α strongly inhibited the induction of all genes examined, leaving only a transient pulse of expression for immediate early genes. This is in complete agreement with the predicted single cycle of p65 activity followed by nuclear sequestration of p65 by IκBα, and the reduced genomic interaction observed in the line FRAP assay.

On the contrary, CHX co-treatment dramatically exaggerated the induction profiles of most genes, with its strongest effect on early genes for which the transcriptional output increased by 5∼50 fold (Fig. 6). The enhanced transcription is again consistent with the model prediction and increased chromatin residence time of p65 in living cells for this condition.

Thus, one signaling cycle or constant action of NF-κB from LMB or CHX, respectively, induced opposite functional consequences: premature termination or uncontrolled accumulation of target gene transcripts. Our data also imply that oscillatory activity of NF-κB strongly constrains the expression of the immediate early genes. The dramatic difference between TNF-α and TNF-α/CHX co-treatment suggests that transcription of NF-κB dependent genes may not occur continuously after TNF-α treatment, but rather in bursts with intermittent ‘off’ periods, echoing periodic p65 cycles.

## Discussion

In this study, we addressed a long-standing debate on the extent of NF-κB oscillation, and we also presented a first comprehensive analysis of the NF-κB signaling system that integrates all time scales of dynamics.

Oscillations of NF-κB have been reported to be strongly damped with one or two cycles from cell population studies [1], while sustained oscillations were detected in single cells expressing fluorescent fusion proteins for p65 [3]. Due to potential artifacts from the additional copies of p65, the single cell results could not be directly applicable to dynamics of endogenous p65 [9]. Because biochemical analysis of asynchronous oscillations is nearly impossible, real-time monitoring of endogenous p65 in single cells was necessary to solve the limitations of the previous approaches. Here we used an ideal physiological NF-κB system—MEF cells with knocked-in GFP-tagged p65—and multiple sensitive optical techniques to visualize the endogenous dynamics. Our work clearly demonstrate the existence of periodic cycles of NF-κB nuclear localization, and establish by line FRAP that NF-κB is active during successive cycles. This finding revises the previous understanding that NF-κB oscillations occur only in cells lacking IκBβ and IκBε but not in wild-type cells [25].

We also examined the dynamics of NF-κB observable in disparate timescales from seconds to hours, and summarize below their functional implications suggested by our data. On the time scale of seconds, NF-κB binding to the chromatin is highly dynamic [19], enabling fast scanning of the genome. This ‘search and activate’ operation has to occur all within a very short timeframe, since the nuclear presence of NF-κB is limited to a brief time window during each oscillatory cycle. The dissociation can occur independently of IκBα and may be mediated by other mechanisms [26], [27], [28].

On the time scale of minutes, NF-κB shuttles continuously between the nucleus and the cytoplasm, allowing for full sampling of the intracellular signaling status and also potentially refreshing itself from inactivating modifications. NF-κB shuttling can proceed without the intervention of IκBs, which has not been recognized previously.

On the scale of hours, oscillations in the nuclear NF-κB level are sustained often up to several cycles, and appear to be determined mostly by the relative strengths of distinct negative feedback loops. Mathematical modeling suggests that the NF-κB network could have selected for non-oscillatory behaviors through the parameter values for the induction kinetics of IκBα and the negative feedback upon IKK. A small fraction of cells that respond to TNF-α with a single cycle in our live imaging further support the idea that the system can avoid oscillation. The sustained NF-κB oscillations seen in most cells despite these alternatives open the possibility that the multiple-cycle action of NF-κB has a functional advantage over non-oscillatory behaviors.

**Figure 7:**
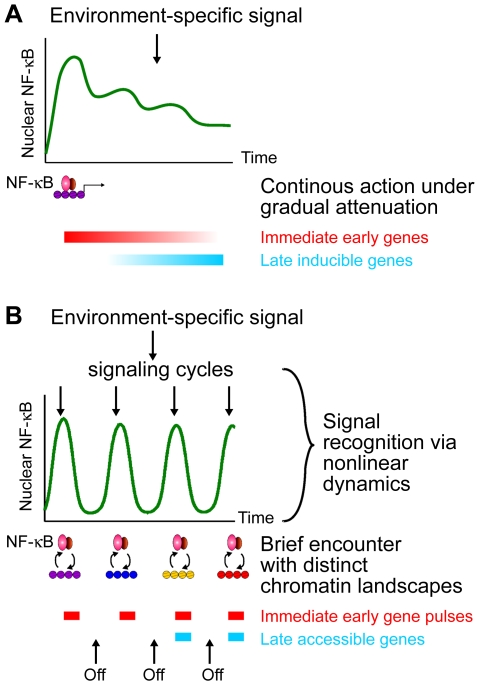
Fine-tuning transcriptional output by NF-κB oscillations. (A) When there are little or no NF-κB oscillations, immediately accessible target genes are continuously induced. Gene-specific mechanisms attenuate the transcription of these genes over time, while a different group of genes become responsive. In this model, overall gene expression kinetics does not critically depend on NF-κB dynamics. (B) Sustained NF-κB oscillations allow only pulses of expression for the immediate early genes, as the transcription factor interacts transiently with the chromatin at discrete times. In later signaling cycles, NF-κB returns with characteristic genome-scanning competency and acts on late-accessible genes, without having accumulated early transcripts at a high level. Therefore, NF-κB oscillations, which are strongly coupled with upstream signaling kinetics, ensure balanced gene expression programs.

Consistent with this hypothesis, p65 mobility by FRAP, reflecting its chromatin residence time, is maintained through the recurrent NF-κB cycles, and is sensitive to perturbations of the natural oscillations. Moreover, such perturbations also alter the transcription of targets, from early to late genes. The late cycles, absent in LMB co-treatment, contribute significantly to late gene expression, while continuous presence of active NF-κB in the nucleus in CHX co-treatment produces exaggerated transcriptional responses. These results suggest that the oscillatory mode of NF-κB action may be a cellular trade-off between efficient pulse expression of immediate early genes and the need for NF-κB to monitor the signaling status for several hours before acting on the modified chromatin landscape [29], [30] (Fig. 7). Limiting the transcription of the immediate genes by periodic inactivity is important in various signaling contexts, especially because many of these encode secreted cytokines and chemokines. Their overproduction would be dangerous in vivo with far-reaching consequences. In summary, fast transient interaction with the chromatin, continuous nucleocytoplasmic shuttling, and long-term oscillations are distinct but inter-dependent mechanisms in functional signaling by NF-κB (compare models in Fig. 7).

The functional relevance of oscillations suggests a previously unappreciated signaling role for the distinct negative feedback loops within the NF-κB regulatory network. Given that negative feedback mechanisms and their relative strengths are major determinants of the oscillatory dynamics, they do not simply function to terminate signaling. Instead, they may promote oscillatory actions of transcription factors for optimal transcriptional responses.

## Materials and Methods

### Cell lines and culture

Cells were cultured in phenol-red free DMEM, supplemented with 10% FBS, 50 µM β-Mercaptoethanol, 1% L-Glutamine, 1% Sodium Pyruvate, 1% non-essential amino acids, and Pen/Strep. Cells were plated on 35 mm coverslip-bottom dishes (MatTek, Ashland, MA) 18∼24 hrs before imaging and the medium was changed to have 2% FBS a few hours before imaging. Reagents were from the following sources: TNF-α (R&D Systems, Minneapolis, MN), Cycloheximide (Sigma, St. Louis, MO), Leptomycin B (LC Laboratories, Woburn, MA). Because both LMB and CHX affect numerous cellular factors that are not specific to NF-κB, we attempted to minimize pleiotropic effects and cytotoxicity by eliminating pre-incubation and by using low drug concentrations (5 nM LMB and 2.5 µg/ml CHX).

### Time lapse microscopy

Live cell imaging of GFP-p65 knock-in MEF was performed using Zeiss LSM 5 Live with an incubation system where cells were stably maintained at 37°C with humidified 5% CO_2_. Time lapse images were acquired at 5 min intervals with a single frame per stage position using 1% of excitation at 489 nm, 63X Plan-Apochromat oil objective (1.4 NA), 0.5 zoom, and a 495 nm long pass filter. Focus was automatically corrected before each time point by a customized the Zeiss Multitime autofocus macro. TNF-α was treated at the second time point by a gentle injection through a tubing. Acquired LSM files were exported to 16 bit TIFF files for further analysis.

### Image quantification and Fourier analysis

16 bit image files were segmented, quantified, and tracked by a custom written MATLAB program that utilizes its Image Processing toolbox. In each time lapse field, we excluded from analysis the cells that moved out of view, died, overlapped with other cells, or divided during the time course. Cell boundaries were identified first automatically by intensity-thresholding then by manual correction. Nuclear boundaries were all manually drawn. The same cells were tracked from the previous time point by finding cells with the closest cell centroids. Background subtracted intensities were used to calculate the ratio of mean nuclear intensity to mean total cellular intensity.

Each time course profile was analyzed by Fast Fourier Transform and the corresponding periodogram was generated to reveal any periodic components. A time course was considered to represent an oscillating p65 if the periodogram had one global maximum or if there was a sharp local maximum at a period less than 5 hours whose value was greater than 70% of the global max. The latter case corresponded to time course profiles where there was a global profile as well as an oscillating component. We are also exploring an alternative method borrowed from audio signal processing, to look for finer temporal components. Some examples are shown in Figure S5.

### Mathematical modeling and computational simulations for multi-parameter analysis

The delay differential equations (DDE) model in [13] was modified by including a term (neg in Fig. S2) to represent the inactivation of IKK by various mechanisms including A20, CYLD, and IKK autophosphorylation. The reference parameter values are listed in Figure S2B [13]. In the multiparameter variation analysis, each rate constant was varied by 2 orders of magnitude around the reference value (from 0.1 to 10 fold) and was randomly combined with others by the Latin Hypercube Sampling method to limit the total computational load at a manageable level. The time delay parameter for IκBα synthesis was constrained to vary between 30 and 55 minutes to avoid an unrealistic range. 1000 samples of parameter combinations were generated and used for DDE simulations. The 1000 time course profiles for free nuclear p65 were classified into eight types using a K-means cluster analysis. All computations were implemented in MATLAB.

### Line FRAP analysis

Line FRAP experiments were performed on a Leica SP5 AOBS confocal equipped with a ArgonPlus Ar-ion laser (220 mW nominal power; power measured at the objective: 11.4 mW for the 488 nm, 3.7 mW for the 596 nm, and 18.1 mW for the 514 nm line), a HCX PL Apo CS 63x oil immersion objective/1.4 NA, and a humidified thermostatic chamber (37°C and 5% CO_2_). The Leica XT-FRAP wizard was used to program the acquisition protocol. Acquisition settings: 16-bit images, 512×512 pixels (59.52 µm×59.52 µm), line size 512×1 pixel, pixel size 116.48 nm, zoom 4x, pinhole Airy 1, 25 Hz with mono-directional scanning and line average = 1; scanning time 40 msec/line. Laser power: For imaging, 5% of the 488 nm single laser line; for bleaching, 100% of the 488, 496, and 514 nm laser lines. Length of bleached line segment: 5 µm. Time course: 20 line scans for pre-bleaching (0.8 s), 5 for bleaching (0.2 s), and 500 for post bleaching (20 s).

Data were doubly normalized as described by Phair et al. (2004) using a region in the nucleus as bleaching reference and one on the glass slide (out of the cytoplasm) for the background (see Fig. 5A). Experiments were repeated four times for each stimulation condition. Non-linear regression fitting of the average curves was obtained using a two-exponentials equation (Y = Ymax1*(1-exp(-K1*X))+Ymax2*(1-exp(-K2*X), half time = 0.69/Ki) with GraphPad Prism 4.0 software. Single representative experiments are shown in Fig. 5.

Statistical significance of separation between different condition groups was assessed by a permutation test based on a 1000 random reshuffling of group labels. P values were calculated from the number of random incidents with mean T statistic greater than the observed. The analysis was implemented in R (‘compareTwoGrowthCurves’ from the library statmod).

### FLIP analysis

FLIP experiments have been performed with the same set-up as for FRAP.

The Leica FRAP wizard was used to program the acquisition protocol. Acquisition settings: 16-bit images, 512×512 pixels (79.4 µm×79.4 µm), pixel size 155 nm, zoom 3x, pinhole Airy 1, 400 Hz in Fly-mode; scanning time 1.317 sec/frame. Laser power: for imaging, 5% of the 488 nm single laser line; for bleaching, 100% of the 488 nm laser line. Bleached region: a round area of 8 µm in diameter in the cytoplasm. Time course: 5 scans for pre-bleaching and 60 scans of bleaching every 10 seconds. Cells were stimulated with TNF-α alone or in co-treatment with LMB or CHX as described above and FLIP acquisitions started 25 minutes later. Data were singly normalized as described by Phair et al. (2004) using a second unbleached cell as “bleaching during imaging” reference and one on the glass slide (out of the cytoplasm) for the background. Experiments were repeated three times.

### Quantitative real-time PCR

p65 knock-in fibroblasts were cultured in 35 mm dishes and treated as described in Figures 6. RNA was prepared with the Illustra RNAspin mini Kit (GE Healthcare). cDNA was synthesized with SuperscriptII RT (Invitrogen). PCR reactions were performed with the LightCycler 480 SYBR Green Master kit on the LightCycler480 Instrument (Roche). Relative quantification (with efficiency correction) of gene transcription was carried out with the software included in the LightCycler480 package. Experiments were repeated three times and representative experiments are shown in Figures 6. Error bars represent standard deviations. Primer sequences are available upon request.

## Supporting Information

Figure S1 Time lapse imaging data quantification. (A) The example shows a time lapse image with boundaries identified by our image segmentation. (B) The quantification of the complete time lapse data for the same field of cells in (A). Cells were treated with 10 ng/ml TNF-α and imaged overnight. The number on top right denotes the cell label from (A). The time lapse movie is provided as Video S1.
(0.58 MB DOC)

Figure S2 Mathematical modeling and simulations for the core NF-κB network dynamics. (A) The 9-variable, 18-parameter delay differential equations model was adapted from [Sung et al. 2004 Mol. Pharm.] with the addition of a term that represents the post-stimulus attenuation of IKK activity (‘neg IKK’ for the equation for IKK). For simulating TNF-α treatment, we used stimulus input k(t) which was fixed for our parameter variations to produce a step-like activation of IKK at t = 0. We note here some simplifications implemented in the model. We combined certain multiple biochemical reactions into single terms. The parameter neg represents the rate of inactivation of IKK by multiple mechanisms. The model also simplifies the induced synthesis of IκBα which in fact comprises of transcription, RNA processing and transport, translation and protein folding. All of these are lumped into a single synthesis term with a delay. Similarly, the catalyzed degradation of IκBα initiated by IKK (rate r1) represents a complex series of reactions including phosphorylation at two serines, ubiquitination, and degradation. Such signal induced degradation is distinguished from the basal degradation of free or NF-κB bound IκBα that occur with rate dg1 or dg2. Notation for the molecule concentrations as model variables: NF = NF-κB I = IκBα IKK = the active IKK complex A colon between two variables indicates a complex of the corresponding proteins, and variables with subscript ‘n’ denote nuclear species. (B) The table describes all the molecular processes that are represented in the model and the reference values for the corresponding parameters. All values are from [Hoffmann et al 2002 Science] except for the following: tau, neg, and s are parameters for simplifying terms that represent multiple biochemical processes. Therefore, their values were estimated arbitrarily so that the TNF-α response profile from these reference parameter values is qualitatively similar to that in [Hoffmann et al 2002 Science]. In fact, we did not assume that any of the parameter values were appropriate for our cells, and instead relied on extensive parameter variations to look for all possible behaviors of the dynamical system from different parameter values. (C) The example below illustrates typical plots from model simulations for TNF-α induced p65 oscillations. The graphs show the time courses of free, IκBα-complexed, and total p65 in the nucleus, as predicted by the model. The red curves indicate the three p65 species in the nucleus from a single run of simulation. (D) The time courses of free active IKK for the eight clusters are shown below. Clusters 1 through 8 correspond to those in Figure 3C. (E) The parameters other than s and neg do not correlate with the distinct patterns of p65 dynamics. The plots below show the parameters d1 (NF-κB:IκBα complex dissociation rate) and r1 (IKK phospho-ubiquitin-degradation of IκBα), for the eight clusters in Figure 3C. Plots for all the other parameter pairs look very similar to this.
(3.14 MB DOC)

Figure S3 Magnitudes of first peak response. The magnitude of first peak response was defined as the maximum nuclear p65 level within 1 hr after TNF-α treatment for each time lapse profile. N is the number of cells for each condition. Error bars are S.D. *** indicates a significant difference from TNF-α with p = 10-4 (Student t test).
(0.10 MB DOC)

Figure S4 Control FLIP experiments for the nucleus-to-cytoplasm flux of free NF-κB in Figure 4D. Fluorescence loss in photobleaching (FLIP) was performed with the same protocol as for the experiment in Figure 4D to compare FLIP under different conditions (TNF-α only, TNF-α + CHX or LMB), as well as FLIP during p65 import. A circular spot in the cytoplasm was repeatedly bleached while the nuclear mean intensity was monitored. The plot in A shows the same data as in Figure 4D for comparison, FLIP time courses acquired 25 minutes after treatments, when p65 translocation is almost complete. Fluorescence profiles are the average of at least 4 cells from three independent experiments acquired at 25 min, 40 min and 55 min after treatment. In TNF-α + CHX there is a decrease in nuclear GFP-p65 signal, indicating the presence of p65 export into the cytoplasm. When cells are treated with TNF only, the rate of p65 export to the cytoplasm is slightly faster, possibly indicating a role of IkBα in removing p65 from the nucleus. In TNF + LMB treated cells, the retrograde flux is almost undetectable, as expected. The plot in B shows the FLIP time course during (not after) the massive nuclear translocation of p65 within 25 minutes after TNF-α or TNF-α + CHX co-treatment. Fluorescence profiles are the averages of at least 10 cells from five independent experiments acquired at 10 min after treatment. In TNF-α + CHX treated cells, there is a slight gain of nuclear p65, instead of a loss, due to a greater nuclear influx of p65 molecules from the unbleached cytoplasmic region in comparison to p65 influx from the bleached area. In TNF-α treated cells, there is a net nuclear loss indicating that p65 export is faster than its import. The error bars indicate S.E.
(0.38 MB DOC)

Figure S5 Application of an alternative signal processing algorithm to representative GFP-p65 time course profiles. We tested an analysis method that is widely used in audio signal processing, against various oscillatory profiles of GFP-p65 time courses in response to TNF-α. First, each time course data y (t) ( = nuclear:total ratio of GFP-p65) was adjusted by baseline subtraction and normalization: y_signal (t) = (y(t) - min y(t))/(max y(t) - min y(t)). Zeros were padded to extend toward negative t values. The y_signal (t) was further processed by Hanning window to produce y_hanning (t). Fast Fourier Transform was then applied to y_hanning and its Bode plot in log scales was generated. The plots in the left column show y_signal (t) as solid curves and y_hanning (t) as dashed. The corresponding Bode plots are shown in the right column. Although this analysis may show fine spectral features of the temporal profiles, it seems less obvious how to classify patterns from Bode plots. Nonetheless, at least the dominant periodic components of p65 seem to be revealed by either this method or our original analysis method in Fig. 1D.
(0.26 MB DOC)

Video S1 Live cell imaging of NF-κB dynamics after TNF-α activation. The time lapse data corresponds to Figure S1.
(11.04 MB MOV)

Video S2 A single cell time series of both the images and the quantified nuclear p65 level. The cell on the left of the initial image frame is followed after TNF-α stimulation.
(11.76 MB MOV)
