# Supplementary figures and images for: Sustained Oscillations of NF-κB Produce Distinct Genome Scanning and Gene Expression Profiles
Source: PLoS One. 2009 Sep 29;4(9):e7163. doi: 10.1371/journal.pone.0007163 (PMC2747007; doi:10.1371/journal.pone.0007163)

**Figure S1.**

**
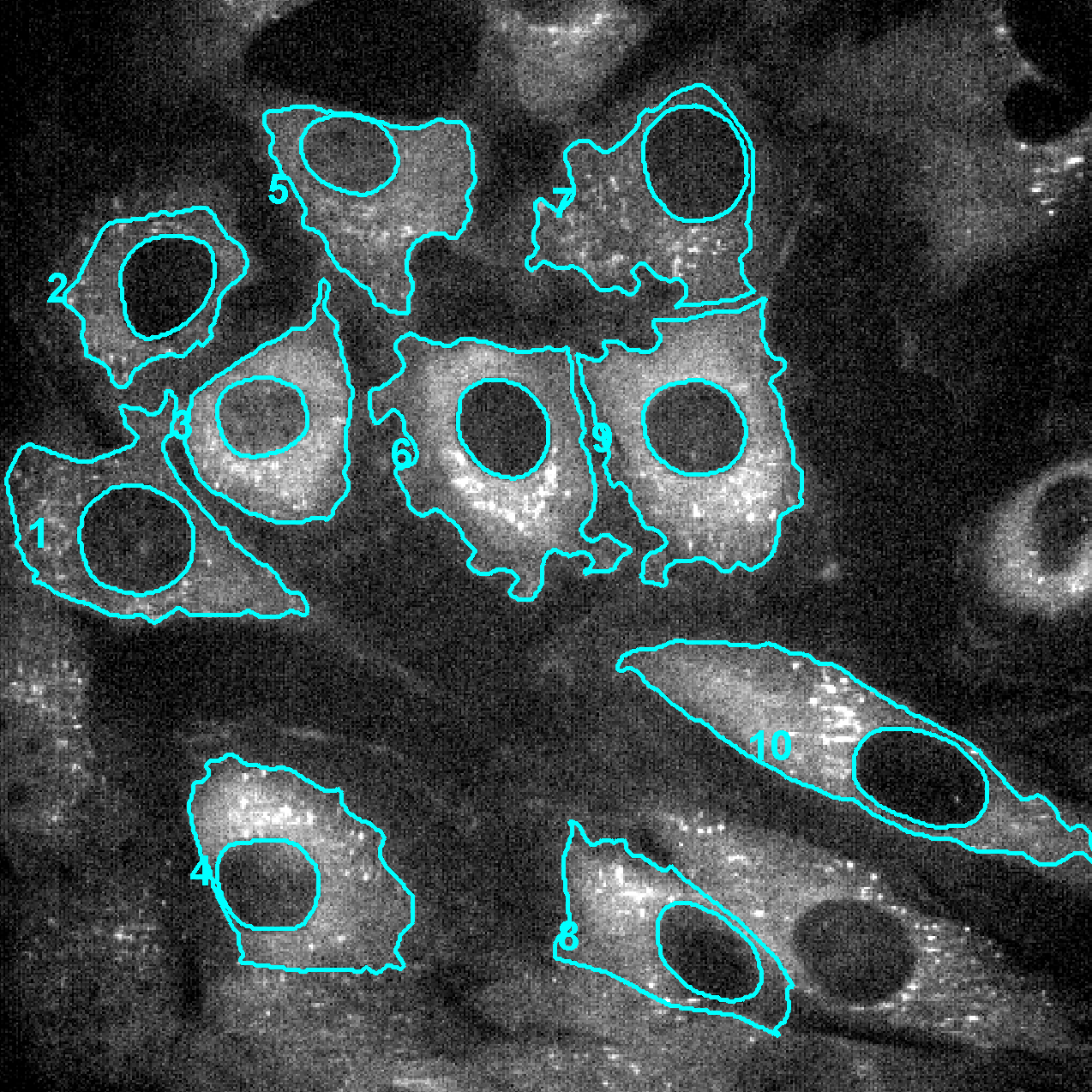

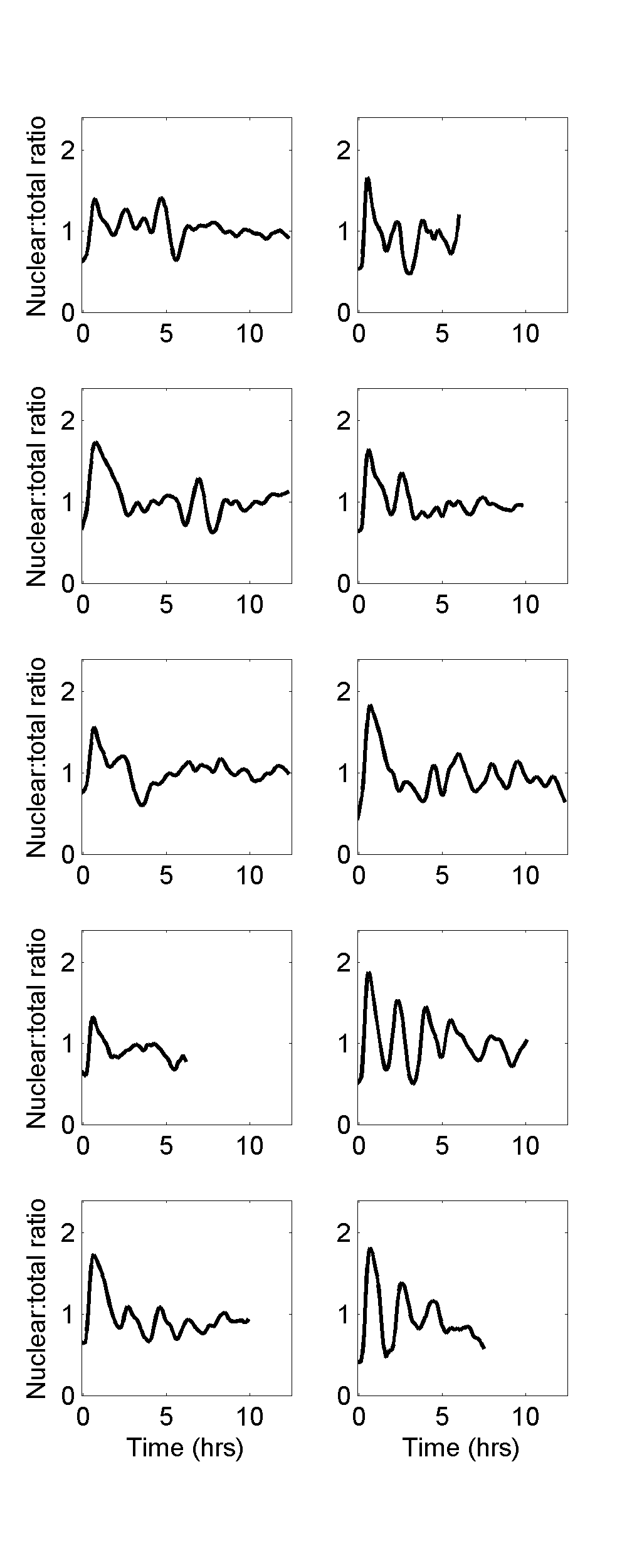
**

**1**

**2**

**3**

**5**

**7**

**9**

**8**

**4**

**6**

**10**

**B**

**A**

Supplement: Figure S1 — Time lapse imaging data quantification. (A) The example shows a time lapse image with boundaries identified by our image segmentation. (B) The quantification of the complete time lapse data for the same field of cells in (A). Cells were treated with 10 ng/ml TNF-α and imaged overnight. The number on top right denotes the cell label from (A). The time lapse movie is provided as Video S1. (0.58 MB DOC) [file pone.0007163.s001.doc]

**Figure S3.**

**
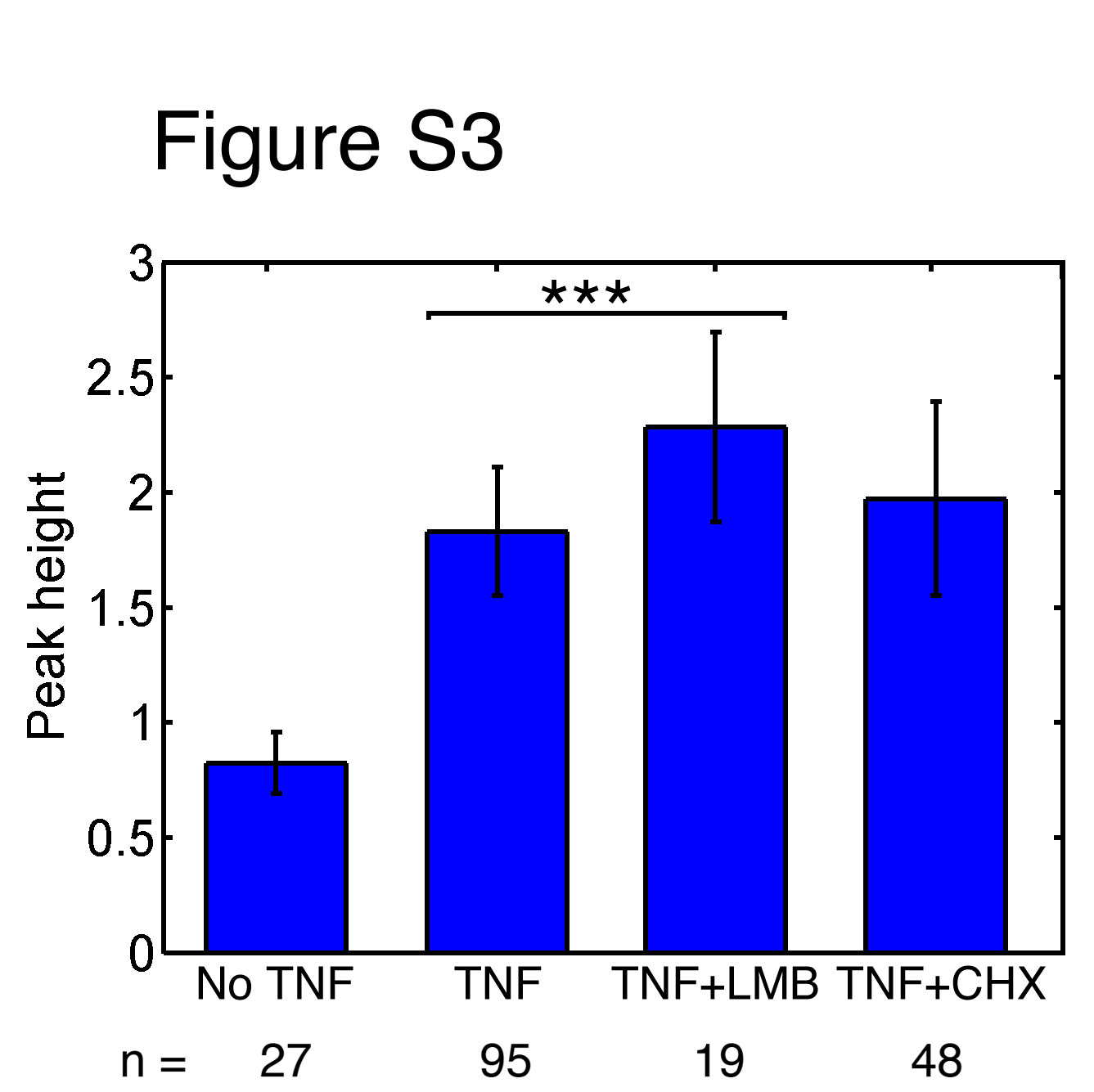
**

Supplement: Figure S3 — Magnitudes of first peak response. The magnitude of first peak response was defined as the maximum nuclear p65 level within 1 hr after TNF-α treatment for each time lapse profile. N is the number of cells for each condition. Error bars are S.D. *** indicates a significant difference from TNF-α with p = 10-4 (Student t test). (0.10 MB DOC) [file pone.0007163.s003.doc]

**Figure S4.**

**
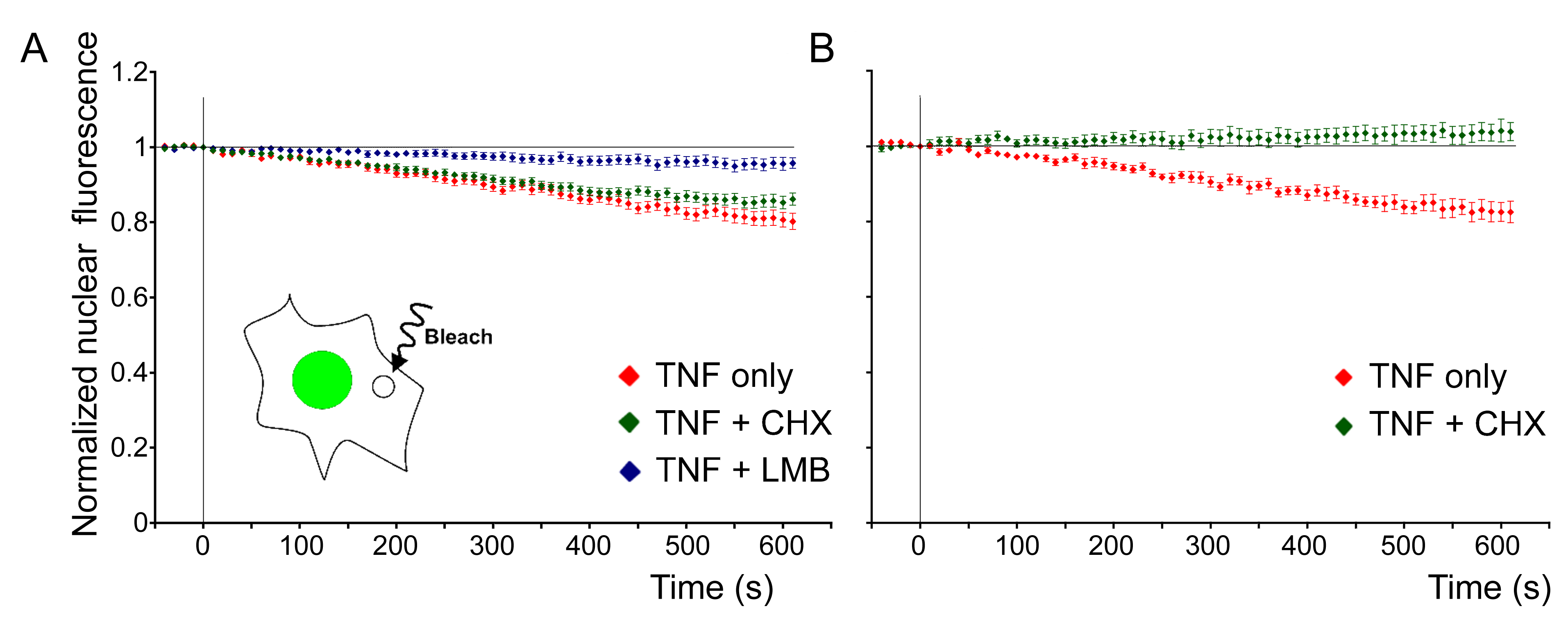
after complete translocation during translocation**

Supplement: Figure S4 — Control FLIP experiments for the nucleus-to-cytoplasm flux of free NF-κB in Figure 4D. Fluorescence loss in photobleaching (FLIP) was performed with the same protocol as for the experiment in Figure 4D to compare FLIP under different conditions (TNF-α only, TNF-α + CHX or LMB), as well as FLIP during p65 import. A circular spot in the cytoplasm was repeatedly bleached while the nuclear mean intensity was monitored. The plot in A shows the same data as in Figure 4D for comparison, FLIP time courses acquired 25 minutes after treatments, when p65 translocation is almost complete. Fluorescence profiles are the average of at least 4 cells from three independent experiments acquired at 25 min, 40 min and 55 min after treatment. In TNF-α + CHX there is a decrease in nuclear GFP-p65 signal, indicating the presence of p65 export into the cytoplasm. When cells are treated with TNF only, the rate of p65 export to the cytoplasm is slightly faster, possibly indicating a role of IkBα in removing p65 from the nucleus. In TNF + LMB treated cells, the retrograde flux is almost undetectable, as expected. The plot in B shows the FLIP time course during (not after) the massive nuclear translocation of p65 within 25 minutes after TNF-α or TNF-α + CHX co-treatment. Fluorescence profiles are the averages of at least 10 cells from five independent experiments acquired at 10 min after treatment. In TNF-α + CHX treated cells, there is a slight gain of nuclear p65, instead of a loss, due to a greater nuclear influx of p65 molecules from the unbleached cytoplasmic region in comparison to p65 influx from the bleached area. In TNF-α treated cells, there is a net nuclear loss indicating that p65 export is faster than its import. The error bars indicate S.E. (0.38 MB DOC) [file pone.0007163.s004.doc]

**Figure S5.**

**
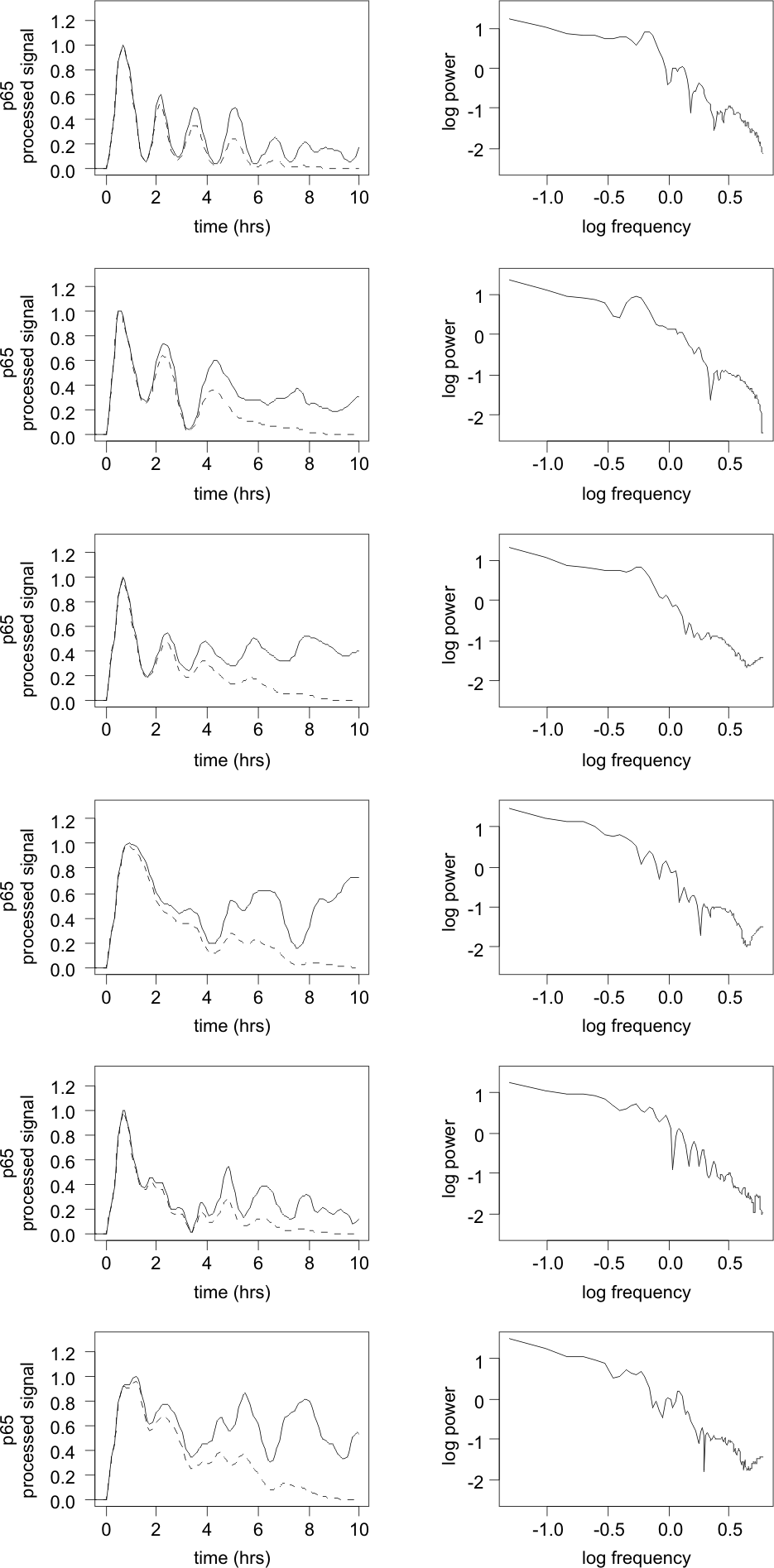
**

Supplement: Figure S5 — Application of an alternative signal processing algorithm to representative GFP-p65 time course profiles. We tested an analysis method that is widely used in audio signal processing, against various oscillatory profiles of GFP-p65 time courses in response to TNF-α. First, each time course data y (t) ( = nuclear:total ratio of GFP-p65) was adjusted by baseline subtraction and normalization: y_signal (t) = (y(t) - min y(t))/(max y(t) - min y(t)). Zeros were padded to extend toward negative t values. The y_signal (t) was further processed by Hanning window to produce y_hanning (t). Fast Fourier Transform was then applied to y_hanning and its Bode plot in log scales was generated. The plots in the left column show y_signal (t) as solid curves and y_hanning (t) as dashed. The corresponding Bode plots are shown in the right column. Although this analysis may show fine spectral features of the temporal profiles, it seems less obvious how to classify patterns from Bode plots. Nonetheless, at least the dominant periodic components of p65 seem to be revealed by either this method or our original analysis method in Fig. 1D. (0.26 MB DOC) [file pone.0007163.s005.doc]
