# Supplementary material for: Sustained Oscillations of NF-κB Produce Distinct Genome Scanning and Gene Expression Profiles
Source: PLoS One. 2009 Sep 29;4(9):e7163. doi: 10.1371/journal.pone.0007163 (PMC2747007; doi:10.1371/journal.pone.0007163)
Supplement: Figure S2 — Mathematical modeling and simulations for the core NF-κB network dynamics. (A) The 9-variable, 18-parameter delay differential equations model was adapted from [Sung et al. 2004 Mol. Pharm.] with the addition of a term that represents the post-stimulus attenuation of IKK activity (‘neg IKK’ for the equation for IKK). For simulating TNF-α treatment, we used stimulus input k(t) which was fixed for our parameter variations to produce a step-like activation of IKK at t = 0. We note here some simplifications implemented in the model. We combined certain multiple biochemical reactions into single terms. The parameter neg represents the rate of inactivation of IKK by multiple mechanisms. The model also simplifies the induced synthesis of IκBα which in fact comprises of transcription, RNA processing and transport, translation and protein folding. All of these are lumped into a single synthesis term with a delay. Similarly, the catalyzed degradation of IκBα initiated by IKK (rate r1) represents a complex series of reactions including phosphorylation at two serines, ubiquitination, and degradation. Such signal induced degradation is distinguished from the basal degradation of free or NF-κB bound IκBα that occur with rate dg1 or dg2. Notation for the molecule concentrations as model variables: NF = NF-κB I = IκBα IKK = the active IKK complex A colon between two variables indicates a complex of the corresponding proteins, and variables with subscript ‘n’ denote nuclear species. (B) The table describes all the molecular processes that are represented in the model and the reference values for the corresponding parameters. All values are from [Hoffmann et al 2002 Science] except for the following: tau, neg, and s are parameters for simplifying terms that represent multiple biochemical processes. Therefore, their values were estimated arbitrarily so that the TNF-α response profile from these reference parameter values is qualitatively similar to that in [Hoffmann et al 2002 Science [file pone.0007163.s002.doc]

**Figure S2.**

**(A)**

**
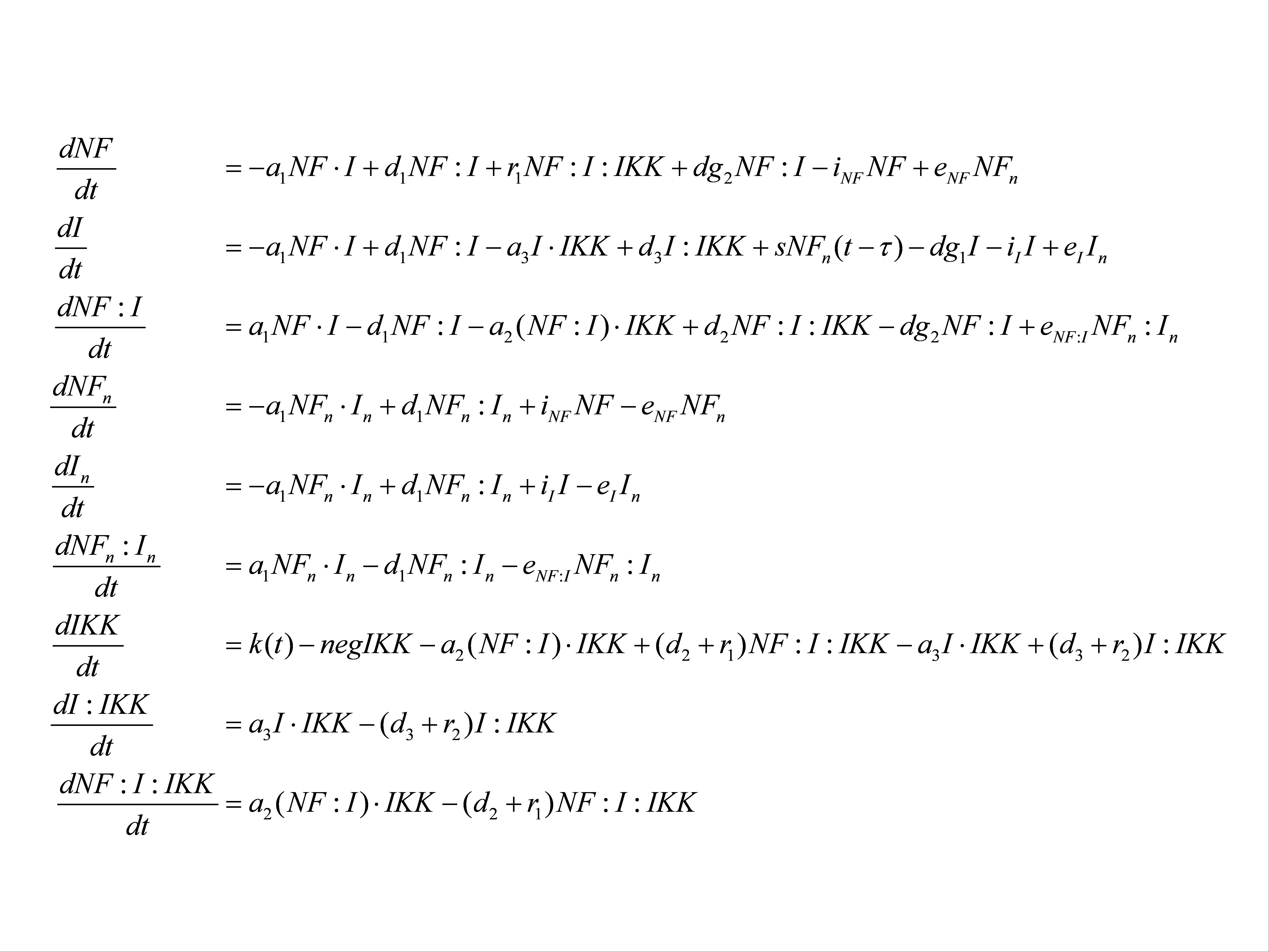
**

**(B)**

| Parameter | Reaction type | Biochemical reaction | Reference value | Unit |
| --- | --- | --- | --- | --- |
| a_1_ | complex formation | NF + I -> NF:I | 30 | µM^-1^ min^-1^ |
| a_2_ | complex formation | NF:I + IKK -> NF:I:IKK | 11.1 | µM^-1^ min^-1^ |
| a_3_ | complex formation | I + IKK -> I:IKK | 1.38 | µM^-1^ min^-1^ |
| d_1_ | dissociation | NF + I <- NF:I | 0.03 | min^-1^ |
| d_2_ | dissociation | NF:I + IKK <- NF:I:IKK | 0.075 | min^-1^ |
| d_3_ | dissociation | I + IKK <- I:IKK | 0.075 | min^-1^ |
| dg_1_ | degradation | I -> 0 | 0.006 | min^-1^ |
| dg_2_ | degradation | NF:I -> NF | 0.0013 | min^-1^ |
| e_NF_ | transport | NF_n_ -> NF | 0.0048 | min^-1^ |
| e_I_ | transport | I_n_ -> I | 0.025 | min^-1^ |
| e_NF:I_ | transport | NF_n_:I_n_ -> NF:I | 0.84 | min^-1^ |
| i_NF_ | transport | NF -> NF_n_ | 5.4 | min^-1^ |
| i_I_ | transport | I -> I_n_ | 0.05 | min^-1^ |
| τ | synthesis | NF_n_ -> NF_n_ + I | 40 | min |
| neg | inactivation | IKK -> 0 | 0.002 | min^-1^ |
| r_1_ | catalyzed degradation | NF:I:IKK -> NF + IKK | 11.1 | min^-1^ |
| r_2_ | catalyzed degradation | I:IKK -> IKK | 2.22 | min^-1^ |
| s | synthesis | NF_n_ -> NF_n_ + I | 0.24 | min^-1^ |

**(C)**

**
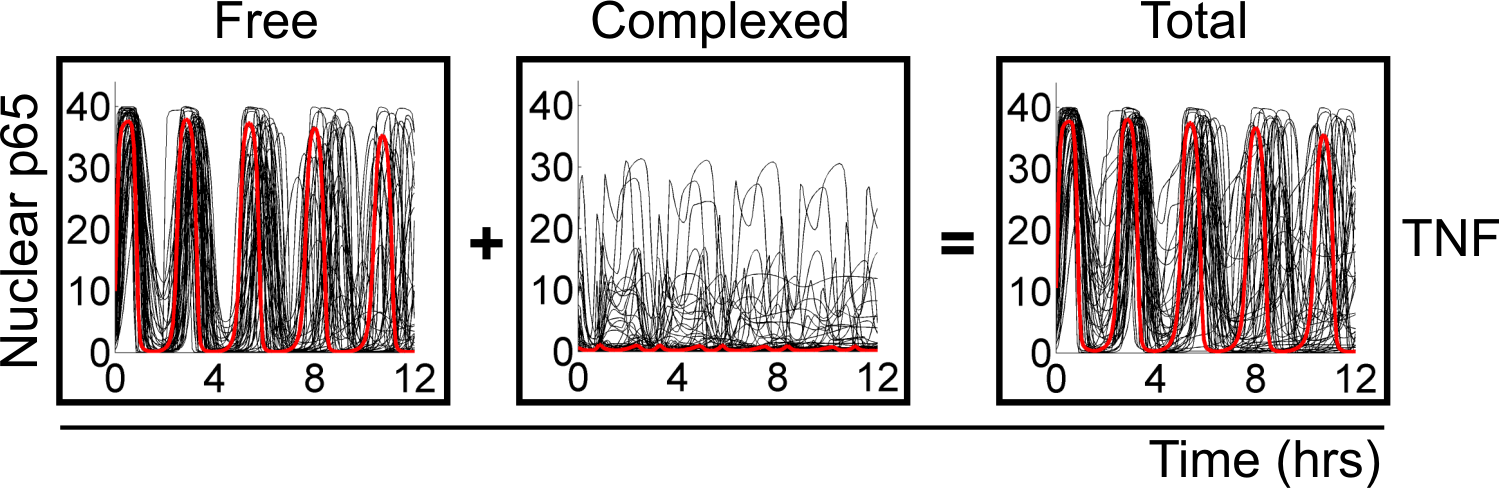
**

**(D)**

**
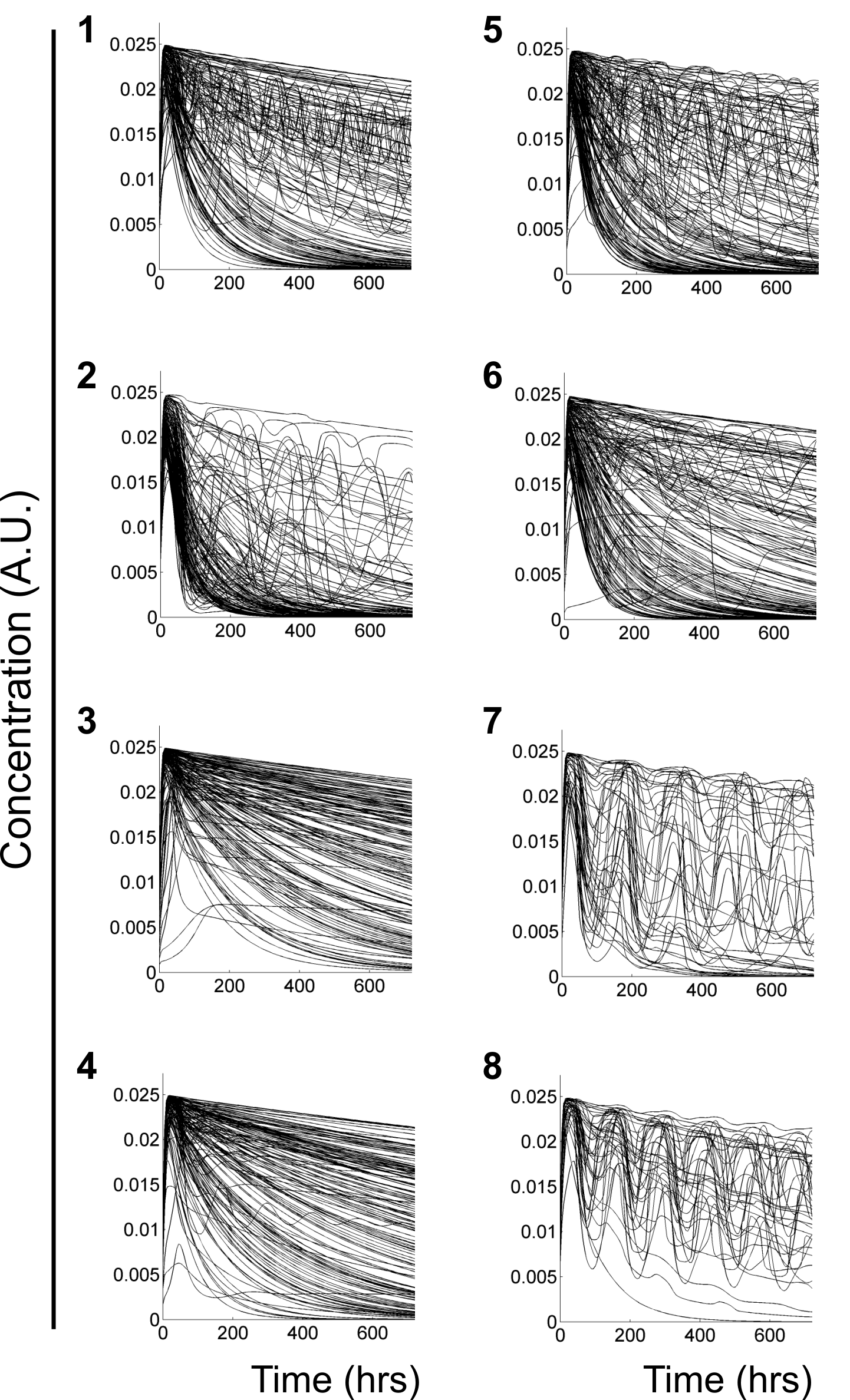
**

**(E)**

**
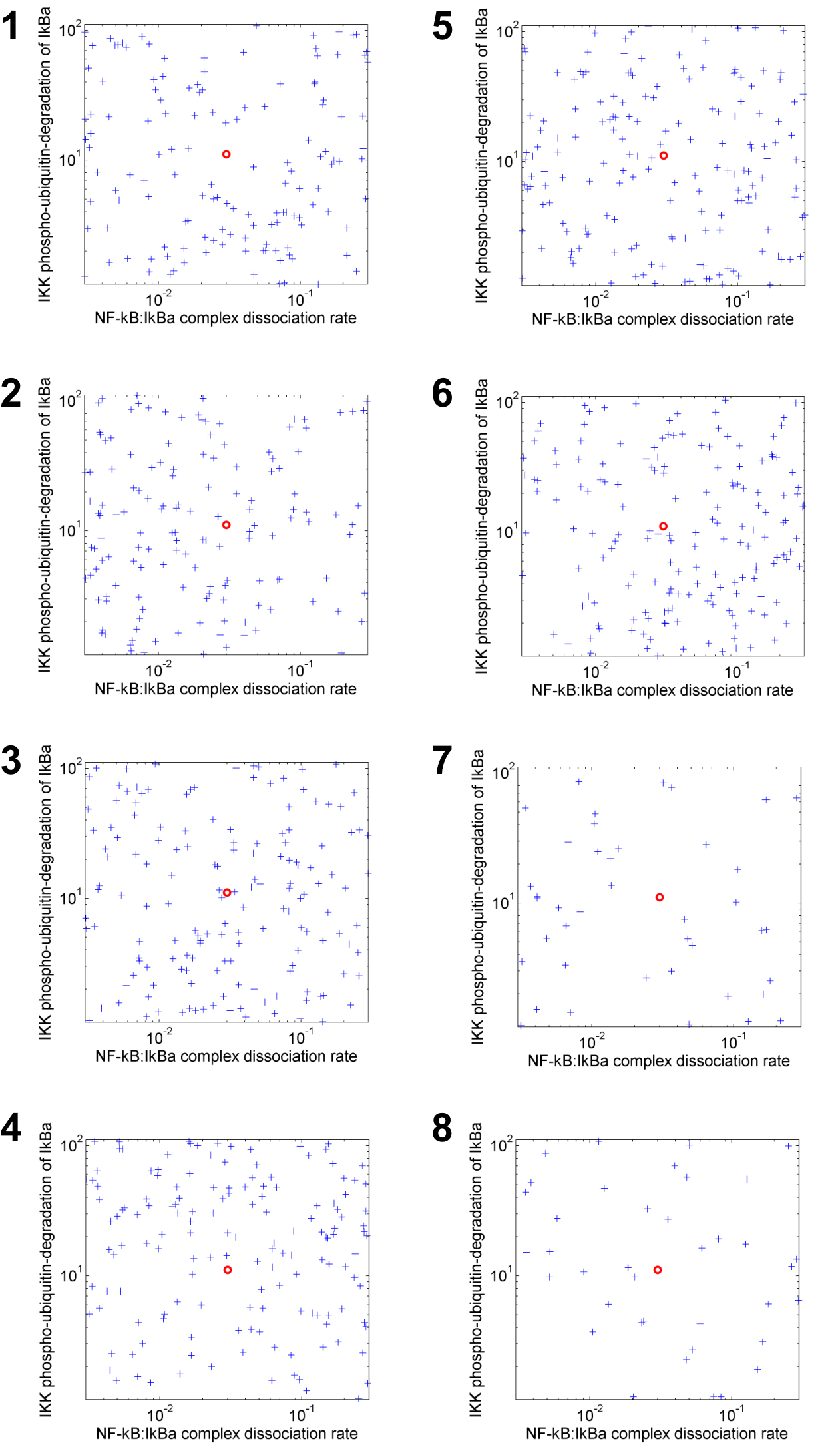
**
